# Hand, Foot, and Mouth Disease as Differential Diagnosis of Monkeypox, Germany, August 2022

**DOI:** 10.3201/eid2812.221487

**Published:** 2022-12

**Authors:** Anahita Fathi, Stefan Schmiedel

**Affiliations:** University Medical Center Hamburg-Eppendorf, Hamburg, Germany (A. Fathi, S. Schmiedel);; Bernhard Nocht Institute for Tropical Medicine, Hamburg (A. Fathi);; German Center for Infection Research, Partner Site Hamburg-Lübeck-Borstel-Riems, Hamburg (A. Fathi, S. Schmiedel)

**Keywords:** monkeypox, MPX, coxsackievirus, enterovirus, hand foot and mouth disease, viruses, sexually transmitted infections, Germany

**To the Editor:** Lewis et al. recommend considering hand, foot, and mouth disease (HFMD) as a differential diagnosis for monkeypox on the basis of a series of 9 patients from Argentina and Bolivia with suspicion of monkeypox, of which 3/9 patients had laboratory-confirmed monkeypox and 4/9 patients had HFMD (*1*). HFMD is common in young children worldwide. Symptoms are usually mild and transient and consist of influenza-like illness, oral sores or pustules, and a palmar and plantar rash (*2*). However, reports about atypical HFMD, which is characterized by severe symptoms, unusual localization of the rash, and occurrence in immunocompetent adults, have recently increased.

Like HFMD, monkeypox often clinically manifests with influenza-like symptoms and a pustular rash (*3*). As of September 2022, >3,500 cases have been reported in Germany (*4*), and differential diagnosis and testing has become increasingly necessary.

We report a case of a 20-year-old man who sought evaluation for monkeypox at University Medical Center Hamburg-Eppendorf (Germany). Two days before, he began experiencing myalgias and fever, followed by a generalized rash with painful pustular lesions on the arms, hands, feet, mouth, scalp, and anus (Figure). He was taking HIV preexposure prophylaxis but had no concurrent conditions. He reported sexual contact with 2 male partners in the 14 days before symptom onset and had regularly visited his family in the previous 29 days, during which time multiple family members experienced influenza-like symptoms and a rash. The patient had called an urgent care provider the day before our evaluation and had been placed under quarantine for suspicion of monkeypox because of his clinical manifestations and medical history.

**Figure:**
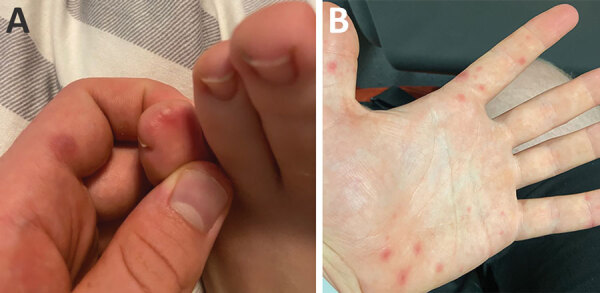
Painful pustular lesions on left foot (A) and hand (B) of a 20-year-old man 1 day after onset of influenza-like symptoms, Germany. PCR results were positive for enterovirus but negative for orthopoxvirus, confirming hand, foot, and mouth disease rather than monkeypox.

We swabbed anal, oral, and skin lesions and assessed the specimens for orthopoxvirus and enterovirus nucleic acids by PCR, which was positive for enterovirus but negative for orthopoxvirus, confirming HFMD. In conclusion, we support the suggestion to consider atypical HFMD as a differential diagnosis of monkeypox.
